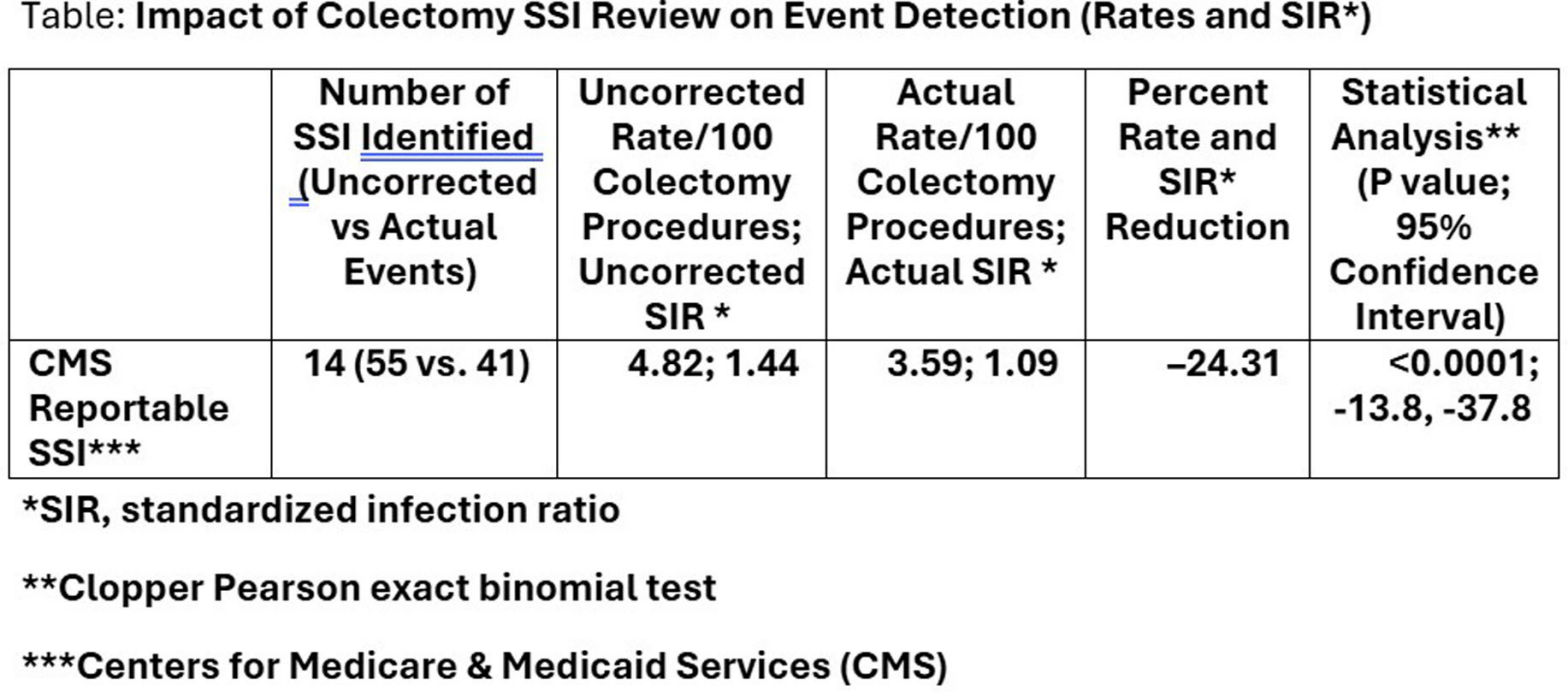# 91 “Providing Us With The Confidence To Be Able To Say No”: Lessons From A Nationwide Antibiotic Stewardship Intervention in Telemedicine

**DOI:** 10.1017/ash.2026.10716

**Published:** 2026-06-23

**Authors:** John Horton, Cheryl Kieta, Tiffany LaFontaine, Xiaogang Wu, Rachel Graham, Werner Bischoff

**Affiliations:** 1 Wake Forest University; 2 Atrium Health Wake Forest Baptist; 3 Advocate Health; 4 AHWFBH

## Abstract

**Introduction:** Surgical site infections (SSI) account for 20% of all healthcare-associated infections (HAIs). SSI are associated with increased patient mortality, morbidity, length of hospitalization and risk of readmission with an associated total healthcare cost of $3.3 billion annually. Of the reported SSIs, colorectal (COLO) surgeries have been among the highest risk procedures. A crucial element to prevent HAI is the accurate identification of true events. This allows for comparison of facility-specific performance metrics with peer groups and the development and implementation of targeted interventions to reduce SSI burden. In this abstract, we assessed the impact of multidisciplinary intervention to accurately identify, and document visualized infections on SSI rates and Standardized Infection Ratios (SIR) at a tertiary care teaching facility. **Methods:** A quasi-experimental study was conducted from 02/01/2024 to 06/30/2025 to improve the accuracy of operative note documentation of visualized infections. Indicators to evaluate possible SSI events included readmission(s), chief complaint, surgical logs, diagnostic codes, antibiotic use, lab/culture data and keywords that may indicate a surgical site infection. Subsequently identified procedures were evaluated by the Surveillance Infection Preventionist team, the Medical Director of Infection Prevention, and the Surgical Quality Officer. If indicated, the operating surgeon was consulted to determine whether visualized infection criteria were met and/or correctly documented. Turnaround time for case reviews was determined. We compared the observed SSI rates and Standardized Infection Ratios (SIR) with the uncorrected rates and SIR accounting for the clarified events. **Results:** Of 1,142 procedures, 55 were initially identified as COLO SSI. After multidisciplinary review, 12 (22%) of the 55 were found to be present at the time of surgery (PATOS). An additional two events (4%) were determined to not meet SSI criteria. The table summarizes the results of the review process showing a statistically significant correction in SSI event rates from 4.82/100 surgeries to 3.59/100 surgeries (p<0.0001). The turnaround time from identification of a potential SSI event to final determination was four days (range: 1-6 days). **Conclusion:** Surgical site infection events are critical quality outcome measures for both regulatory and reputational agencies. However, the misclassification of SSIs, such as observed in our study, can create a misleading picture of institutional performance, divert attention from preventable cases, and undermine confidence in surveillance systems. Our multidisciplinary approach ensures a more accurate identification of SSI events. This method can serve as a model for enhancing surveillance accuracy and reliability across healthcare settings.

**Figure:**